# Racial Stress, Social Support, and Racial Socialization Among Rural Black Mothers: Associations With Preschoolers' Executive Functioning

**DOI:** 10.1111/famp.70068

**Published:** 2025-08-31

**Authors:** Qiong Wu, Xinyun Kaikai Zhang, Chioma Opara, Ming Cui, Penny Ralston

**Affiliations:** ^1^ Department of Human Development and Family Science Florida State University Tallahassee Florida USA

**Keywords:** Black families, executive functioning, racial socialization, racial stress, social support

## Abstract

Racial stress significantly affects Black mothers and their children. To cope, mothers often use racial socialization strategies, such as preparation for bias, cultural socialization, and promotion of mistrust, to help their children manage race‐related stress. Guided by the Racial Encounter Coping Appraisal and Socialization Theory, this study investigated the relations among racial stress, social support, and racial socialization among low‐income, rural Black mothers and their associations with preschoolers' executive functioning. The study utilized data from 437 Black mothers and their preschool‐aged children. Mothers reported their social support over 3 years, as well as racial stress and racial socialization practices. Preschoolers' executive functioning was assessed using a series of laboratory tasks. Findings from a path model indicated that both racial stress and social support predicted the use of racial socialization strategies. Notably, social support was linked to higher cultural socialization under high racial stress. Additionally, maternal racial stress moderated the relations between racial socialization and child executive functioning: preparation for bias was associated with lower executive functioning under high maternal racial stress, and promotion of mistrust was linked with lower executive functioning under low stress. The study highlights the importance of age‐appropriate, context‐sensitive racial socialization strategies and the need for supportive networks to enhance positive outcomes for Black mothers and children in racially stressful environments.

## 1 Introduction

Racial stress is defined as the psychological and physiological strain that individuals experience because of racism and racial discrimination. It arises when people encounter or anticipate racially charged experiences that challenge their sense of safety, identity, or well‐being (Harrell 2000). In the U.S., individuals identifying as Black commonly perceive racial stress, and the stressful events can occur at interpersonal, institutional, and systemic levels (Harrell 2000; Umaña‐Taylor and Hill 2020). Over 50% of Black mothers with preschoolers reported at least one discriminatory racial event (Condon et al. 2022), posing significant challenges to Black mothers' mental and physical health (Condon et al. 2022; Harrell 2000).

In navigating racial stress, Black mothers typically rely on social support as a coping resource (Bryant et al. 2024; Hudson et al. 2016). They have also learned the necessity to prepare their children for such stressful experiences and may use racial socialization as a parenting practice to teach their children to process and manage race‐related stress (Anderson and Stevenson 2019). However, despite growing interest in these processes, little is known about how maternal social support, racial stress, and racial socialization jointly relate to children's executive functioning. To address the gaps in the literature, the current study sought to examine how maternal social support, racial stress, and racial socialization strategies were related to preschoolers' executive functioning among a sample of low‐income, rural Black families.

### 1.1 Executive Functioning Development in a Low‐Income, Rural Environment

Executive functioning (EF), core cognitive processes to plan, organize, regulate attention and emotion, and achieve goals (Bernier et al. 2010), is essential for forming adaptive behavior, emotional well‐being, academic success, and peer relationships (e.g., Becker et al. 2014; Caporaso et al. 2019). During early childhood, children typically show notable development in key areas of EF: *inhibitory control* (the ability to suppress a dominant response in favor of a less automatic one), *attentional flexibility* (the capacity to shift focus between different mental tasks), and *working memory* (the ability to hold and manipulate information over short periods; Willoughby et al. 2010). Given the vast development of EF in early childhood, it is particularly malleable and thus susceptible to the adverse effects of chronic stress and environmental adversity experienced at this age.

Theoretical frameworks on EF development typically emphasize an interplay between parenting and contextual stress in the proximal environment (e.g., Blair and Raver 2015; Bridgett et al. 2015). For Black preschoolers living in low‐income, rural environments, EF development may be particularly compromised by a convergence of structural and psychosocial stressors. In rural, under‐resourced settings, families often face economic hardship, limited access to quality early education, and fewer community supports, all of which can reduce opportunities for cognitive stimulation that are needed for EF development (Blair and Raver 2015). For example, Tine (2014) found that low‐income rural children showed lower EF scores (particularly working memory) compared to high‐income rural children and urban children. Additionally, particular stressors (such as inadequate learning materials) were found to link with lower EF among preschoolers in only low‐income but not high‐income rural families (Murphy et al. 2022).

Additionally, Black families may experience racial discrimination and systemic inequities that contribute to elevated parental stress. High maternal racial stress may contribute to a more stressful home environment, increasing distress levels in children. Children may also internalize their mothers' discrimination experiences as likely experiences for themselves in the future. The perceived likelihood of experiencing racism may negatively impact their neurodevelopment and subsequently their ability to focus, regulate emotions, and process information efficiently, impairing their EF development (Heard‐Garris et al. 2018). Chronic exposure to stress can particularly dysregulate the child's stress response system, particularly the hypothalamic–pituitary–adrenal (HPA) axis, thereby affecting the maturation of brain regions responsible for EF, such as the prefrontal cortex (Bernier et al. 2010; Bridgett et al. 2015). Indirect experiences of parental racial stress have been found to link with physical health and behavioral issues (Heard‐Garris et al. 2018) and disruptions in EF among preschoolers (Barbee et al. 2025). Given the important role of EF in children's academic and socioemotional development, studying the relations between race‐related stressors and EF has significant implications, as compromised EF may affect low‐income Black children's educational attainment, as well as physical and mental health, perpetuating poverty and health issues over generations.

### 1.2 Racial Encounter Coping Appraisal and Socialization Theory

To conceptualize racial stress, the Racial Encounter Coping Appraisal and Socialization Theory (RECAST; Anderson and Stevenson 2019; Stevenson 2014) draws upon the transactional model of stress and coping (TMSC; Lazarus and Folkman 1984) and conceptualizes stress and coping in the context of racism at the family level. The RECAST further integrates the parenting literature and emphasizes the critical role of racial socialization in reducing stress associated with discriminatory racial encounters.

The original TMSC focuses on the interactive nature of a perceived stressor and available coping resources for the well‐being of individuals. The RECAST furthers this conceptualization and points out that the perceived racial stress and coping resources collectively and interactively inform individual well‐being. That is, parents must possess the resources to manage their own stress before effectively supporting their children through coping with race‐related stress (Anderson and Stevenson 2019).

Social support is recognized as a crucial resource factor for low‐SES Black mothers in navigating stress caused by discrimination and prejudice (Bryant et al. 2024; Hudson et al. 2016). Black mothers embrace unique cultural values that could help them adapt and survive in the face of contextual challenges such as racism (Umaña‐Taylor and Hill 2020). More specifically, low‐SES Black mothers often rely on extended families, kinships, friendships, and religious communities to form a support network that helps them manage the multiple demands of their lives (Bryant et al. 2024; Hudson et al. 2016). It is especially important for Black mothers to receive such support in rearing a young child (Bryant et al. 2024; Hudson et al. 2016). Social support thus serves as an important resource that provides Black mothers with tools to navigate racial stress and parenting demands and, more importantly, engage in more effective parenting strategies.

The RECAST additionally emphasizes that the balance between stress and resources extends to their children, forming intergenerational collaboration in coping with racial stress. The RECAST considers *racial socialization*, or parent–child communication about race‐related information, as a dyadic coping process between family members (e.g., parent to child) and serves as a family‐level resource for children to manage race‐related stress (Anderson and Stevenson 2019). This framework suggests that explicit and practiced racial socialization could enhance children's competence in employing coping strategies, leading to improved long‐term well‐being, especially when there are greater environmental demands (i.e., racial stress; Anderson and Stevenson 2019). This coincides with the theories emphasizing an interaction between parenting and contextual stress, such as racial stress, for EF development (e.g., Blair and Raver 2015; Bridgett et al. 2015).

Collectively, the RECAST serves as an overarching framework that bridges racial stress as an external stressor and racial socialization as a parental response to cope with contextual demands. However, evidence empirically testing this framework has been lacking. Most current evidence also centers on adolescents and youth, with little research investigating early childhood outcomes, highlighting a need for such investigations.

### 1.3 Racial Socialization Strategies

Black mothers use racial socialization strategies to convey messages about race and ethnicity to their children (Anderson and Stevenson 2019; Hughes et al. 2006; Umaña‐Taylor and Hill 2020). This practice extends to younger children, as children can detect racial cues and form racial attitudes even before age 3 (e.g., Caughy et al. 2002; Edwards and Few‐Demo 2016). Several common strategies are crucial for helping children understand their racial identity and cope with racial bias. First, *preparation for bias* involves warning children of potential racial discrimination and teaching them how to cope with it. Mothers might discuss historical and contemporary examples of racism and provide coping strategies. Studies generally indicate that preparation for bias can help children develop resilience and better manage stress related to discrimination (Evans et al. 2012; Hughes et al. 2006). However, it may also be important for mothers to tailor the content and complexity of discussions to the child's developmental stage and balance these conversations with positive messages (Edwards and Few‐Demo 2016). Preschoolers may not fully grasp the concepts of racism, and preparation for racism may have negative outcomes. For example, in a sample of low‐income Black families, parents' preparation for bias and experienced racism collectively were linked with greater child internalizing behaviors between ages 6 and 7 (Osborne et al. 2021).

Second, *cultural socialization* involves teaching children about their cultural upbringing and instilling pride in their racial or ethnic background. It includes sharing cultural traditions, stories, and practices. Cultural socialization can enhance children's self‐esteem and provide a strong sense of identity (Hughes et al. 2006; Wang et al. 2020). Black mothers favored and intentionally used cultural socialization with preschoolers as a developmentally appropriate approach, especially when they recognized the timing of readiness for children's social understanding (Edwards and Few‐Demo 2016). Aguayo et al. (2021) observed cultural socialization conversations between parents and preschoolers, which were linked to positive outcomes, such as increased self‐esteem and better social skills. Cultural socialization was positively related to children's academic performance, motivation, and engagement (Wang et al. 2020), and reading comprehension in middle childhood among Black children (Banerjee et al. 2011). For preschoolers, early exposure to cultural knowledge and practices was associated with better cognitive development, readiness for school (Wang et al. 2020), effortful control (Derlan et al. 2018), as well as lower behavioral problems (Caughy et al. 2002).

Last, parental *promotion of mistrust* entails teaching children to be cautious of other racial groups due to potential bias or discrimination. Although this strategy can protect children from harm, it may also lead to increased anxiety and social withdrawal (Hughes et al. 2006; Umaña‐Taylor and Hill 2020). Research on parental promotion of mistrust among preschoolers has been limited; one qualitative study revealed that Black parents did not favor this strategy towards their 1–3‐year‐olds (Blanchard et al. 2019). Among Black adolescents, parental promotion of mistrust was linked with anxiety symptoms and conduct problems (Kwon et al. 2022; Saleem et al. 2023). Park et al. (2020) found that paternal promotion of mistrust exacerbated the link between discrimination experiences and depressive symptoms among Mexican‐origin adolescents. These findings indicate that while parental promotion of mistrust aims to protect, it may also increase anxiety and depressive symptoms by heightening awareness of potential threats.

### 1.4 Maternal Racial Stress Moderating Links From Social Support to Racial Socialization

Together, racial stress, which includes experiences of discrimination, microaggressions, and concerns about racial injustice, can shape how Black mothers communicate messages about race and culture to their children (McNeil Smith et al. 2016; Saleem et al. 2020). For example, Black mothers with more racial discrimination experiences tended to emphasize messages about preparation for bias, cultural socialization, and promotion of mistrust (White‐Johnson et al. 2010; Witherspoon et al. 2022).

Beyond the direct effects, racial stress serves as a contextual moderator in the association between resources and racial socialization. According to the RECAST (Anderson and Stevenson 2019), a strong social support network serves as a resource that may strengthen the cultural identity of Black mothers and shape the ways in which mothers engage in more effective racial socialization. Given high racial stress, socially supported Black mothers may especially feel the pressing need to teach their children about race and engage in education about cultural identity.

Past research has also supported the role of racial stress in moderating the link between resource factors, such as social support, and racial socialization. For example, Saleem et al. (2016) found that racial discrimination strengthened the negative effect of neighborhood cohesiveness on the promotion of mistrust among Black parents. This seems to suggest that resource factors such as social support can strengthen parental cultural identity, and they tend to engage in more effective racial socialization in the context of high racial stress. Yet, it would be especially interesting to examine whether Black mothers with strong social support may be more likely to adopt affirming strategies like cultural socialization or rely on fear‐based approaches like the promotion of mistrust in the context of high racial stress.

### 1.5 Maternal Racial Stress Moderating Links From Racial Socialization to Child EF

The RECAST points out the intergenerational process of how maternal racial stress may affect their children by serving as a contextual moderator of maternal racial socialization practices (Anderson and Stevenson 2019). This draws on literature supporting an interaction between parenting and contextual stress predicting EF development (e.g., Blair and Raver 2015; Bridgett et al. 2015). For example, preparation for bias and cultural socialization buffered the negative effects of racial stress on academic achievement among Black adolescents (Banerjee et al. 2018; Neblett et al. 2006) and psychological well‐being (Dunbar et al. 2022; Jiménez and Smalls‐Glover 2023). Espinoza et al. (2016) found that in a sample of Mexican American teens, parents' experiences of racial discrimination intensified the link between parental promotion of mistrust and lower adolescent self‐esteem, and also attenuated the association between parental cultural socialization and higher adolescent self‐esteem. As for preparation for bias, Espinoza et al. (2016) found that only in the context of high parental racial discrimination, preparation for bias was linked with low adolescent self‐esteem. Similarly, low‐income Black parents' experienced racism intensified the relations between preparation for bias and first graders' internalizing behaviors (Osborne et al. 2021).

Based on the evidence, it is speculated that under strain from indirect (parental) experiences with racism, parental messages to prepare for potential race‐based bias might signal more warning than just precaution for children; therefore, intensifying the effects on young children (Osborne et al. 2021). Similarly, the RECAST (Anderson and Stevenson 2019) suggests that in the context of high racial stress, the effects of resources (such as parental cultural socialization) on children's well‐being become more prominent; however, the effects of risk factors (such as parental promotion of mistrust) may also strengthen. The high‐risk nature of racial stress may also transform preparation for bias into a less adaptive socialization strategy.

### 1.6 The Current Study

Guided by the overarching framework of the RECAST (Anderson and Stevenson 2019), the current study examined the multifaceted associations among maternal social support, racial stress, racial socialization strategies, and preschoolers' EF among a sample of low‐income, rural Black families. We investigated the moderating roles of racial stress in the relations between social support and racial socialization strategies and between racial socialization strategies and preschoolers' EF. This study addresses several gaps in previous research. First, the vast evidence on parental racial socialization has focused on adolescents, whereas child outcomes as early as 3 years old have been under‐investigated. Preschoolers are still developing their understanding of social meanings, and possibly due to this, limited evidence suggests that socialization messages (such as preparation for bias) that might benefit older children showed adverse effects on preschoolers (e.g., Osborne et al. 2021). This reveals a key gap concerning the developmental appropriateness of racial socialization strategies within this age group. Second, no known study has examined the effect of maternal socialization on preschoolers' EF, which develops vastly in this age and is critical for lifelong well‐being and success (Bernier et al. 2010). Last, the intergenerational effects of parental racial stress on their children are under‐examined. This is important as many preschoolers spend a significant amount of time at home, and their encounters with racial stress may be less direct but mostly indirect from their mothers.

To address these gaps, this study tested the following hypotheses. First, whereas maternal social support and racial stress would generally predict high usage of racial socialization strategies, maternal racial stress would intensify the relation between maternal social support and racial socialization, such that mothers perceiving more social support would more frequently use preparation for bias, cultural socialization, and promotion of mistrust in the context of high racial stress. Second, maternal racial stress would intensify the association between maternal racial socialization and child EF. In the context of high racial stress, cultural socialization would be associated with better child EF, whereas preparation for bias and promotion of mistrust may be associated with worse child EF.

## 2 Method

### 2.1 Participants and Procedures

This study used data from the Family Life Project (FLP; Vernon‐Feagans et al. 2013). Participants were recruited from hospitals in U.S. regions with high poverty rates. The FLP employed a stratified sampling method, oversampling low‐income and Black families (original *N* = 1292). To qualify for the current study, mothers had to identify as Black, non‐Hispanic, resulting in a sample of 437 mother–child dyads (50.1% girls).

When children were 3 years old, mothers had an average age of 27.35 years (SD = 5.43). About a third of the mothers (36.2%) were married, with another 12.4% cohabiting with unmarried partners. Two‐thirds of the mothers (63.4%) were employed. The mean years of education was 14.34 (some college education; SD = 2.48, range 7–21). The mean family income‐to‐needs ratio was 1.26 (SD = 1.20). Compared to the rest of the FLP sample, mothers in the current sample were more likely to reside in North Carolina (rather than Pennsylvania; *t*(1174) = 30.08, *p* < 0.001), unmarried (*t*(900) = 11.76, *p* < 0.001), younger (*t*(1069) = 8.59, *p* < 0.001), less educated (*t*(1096) = 6.02, *p* < 0.001), and lower‐income (*t*(1074) = 10.58, *p* < 0.001).

Mothers provided research consent for themselves and for their children and reported demographic information at the 2‐month assessment. Mothers reported their perceived social support at 2, 6, 24, and 36 months of child age. At the 36‐month assessment, children's EF was assessed using laboratory tasks, whereas mothers also reported perceived racial stress and racial socialization practices. Ethical approval for the FLP was obtained from the Institutional Review Boards (IRBs) of participating universities. The secondary analyses of the data were approved by the IRB of Florida State University (STUDY00000305, “Parenting and child emotional development”). This study was not preregistered. All procedures performed in studies involving human participants were in accordance with the ethical standards of the institutional and/or national research committee and with the 1964 Helsinki declaration and its later amendments or comparable ethical standards.

### 2.2 Measures

Maternal *social support* was measured at 2, 6, 24, and 36 months using the Questionnaire of Social Support (QSS; Crnic et al. 1983) to evaluate their perceived availability of support across extended family (5 items, e.g., “help that family provides”) and friends (4 items, e.g., “talk with friends on phone”). Satisfaction was rated on a 4‐point scale (1 = *very dissatisfied*, 4 = *very satisfied*). Mean scores were calculated for the 9 items to create a composite score at each time point, and an average score was generated to indicate perceived social support across the first 3 years of postpartum, to reduce measurement error and generate a stable, trait‐like indicator across time (e.g., Conger et al. 2000). Cronbach's αs ranged between 0.79 and 0.84 across four waves, demonstrating acceptable to good reliability.

Maternal *racial socialization* was reported at 36 months by mothers using a 15‐item Racial Socialization measure comprising three subscales: *preparation for bias* (6 items), *cultural socialization* (5 items), and *promotion of mistrust* (4 items). This measure was developed by the FLP team, adapted from the Parents' Racial Socialization Scale by Hughes and Johnson (2001), and designed to measure racial socialization practices among Black mothers. Items ask about messages that the mother may have given the child in the past month. Sample items include “talked with the child about the possibility that some people might treat him/her badly or unfairly because of his/her race” (preparation for bias), “talked to the child about important people or events in the history of your racial group” (cultural socialization), and “encouraged the child to keep his/her distance from kids of a different race or ethnicity than his/hers” (promotion of mistrust). Items were rated based on frequency on a 4‐point scale (1 = *never*, 4 = *more than 5 times*). A mean score was generated, with higher scores indicating more frequent use of the corresponding socialization strategy. Cronbach αs were 0.88 (preparation for bias), 0.83 (cultural socialization), and 0.64 (promotion of mistrust), respectively.

Maternal *racial stress* was assessed at 36 months using a 17‐item Race‐Related Stress Events measure, developed by the FLP team. This measure asked mothers about the stressfulness of race‐related events during the past 5 years, capturing salient, chronic exposure that is relevant to parenting behavior. Sample items include “Gotten into an argument about something racist that happened” and “Been called a racist name,” rated on a 5‐point scale (0 = *did not happen*, 4 = *very stressful*). A total score was generated, with higher scores indicating higher racial stress. This measure had excellent internal consistency; Cronbach α was 0.94.

Children's *executive functioning* was assessed during the 36‐month assessment using a series of tasks. Specifically, *inhibitory control* was measured through three tasks: first, the *Animal Go/No‐Go* task (Durston et al. 2002). Children were given a button to press when an animal image appeared (“go” trials) with varying numbers of times before not pressing the button when a pig was shown (“no‐go” trial, 7 trials, scored). The *Spatial Conflict* task (Gerardi‐Caulton 2000) involved first building a prepotent response when children learned to press a button on the left or right based on the arrow cue, then requiring children to inhibit their learned response by showing the arrows contralaterally (13 trials, scored). The third task, the *Silly Sounds Stroop* task (17 items; Gerstadt et al. 1994), required children to make incongruent sounds (meowing at a dog image and barking at a cat image), assessing their ability to override automatic responses. *Working memory* was evaluated through the *Working Memory Span* task (4 items; Engle et al. 1999), where children first saw a picture of a house and an animal together and then were shown the house alone and asked to recall the animal. Last, *attention shifting* was assessed with the *Something's the Same* task (14 items; Jacques and Zelazo 2001). Children were shown two items that were similar in one dimension (shape, size, or color). They were then presented with a third item, similar to one of the original items in a different dimension, and asked to identify which of the initial items was similar to the third. The five tasks were developmentally appropriate for 3‐ to 5‐year‐olds (Willoughby et al. 2010) and scored using item response theory models, which calculate an expected a posteriori (EAP) score. EAP scores estimate a latent trait, considering both item difficulty and individual ability, and provide a calibrated measure of the likelihood of task success. A composite score was generated for overall EF, with higher scores reflecting better executive function (details in Willoughby et al. 2010).

Covariates include the recruitment site (0 = *North Carolina*, 1 = *Pennsylvania*), child sex (1 = *girl*, 0 = *boy*), and child birth order (1 = *firstborn, 0 = not firstborn*), reported at baseline; and maternal marital status (1 = *married*, 0 = *not married*), cohabitation (1 = *non‐married cohabitating*, 0 = *not cohabitating*), employment (1 = *employed*, 0 = *not employed*), age, years of education, and family income‐to‐needs ratio, all assessed at 36 months.

### 2.3 Analytic Plan

Little's MCAR test revealed that data were missing completely at random, χ^2^/df = 1.79, smaller than 2, which suggested a close fit. Full Information Maximum Likelihood (FIML) estimation was employed to minimize bias in handling missing data (Enders and Bandalos 2001), with robust standard errors (MLR) applied to account for potential skewness.

Analyses were then performed using the *lavaan* package in R, with a path modeling approach. Maternal social support, racial stress, and the interaction between them predicted three racial socialization strategies separately, and then maternal social support and racial stress and their interaction, as well as three racial socialization strategies and their interactions with racial stress, predicted child EF. All variables included in the moderation analyses were mean‐centered. Model fit was assessed using the Root Mean Squared Error of Approximation (RMSEA), Comparative Fit Index (CFI), and standardized root mean squared residual (SRMR). A good model fit was indicated by an RMSEA/SRMR between 0.00 and 0.05 and a CFI between 0.95 and 1.00, while an acceptable fit was indicated by an RMSEA/SRMR of 0.05 to 0.08 and a CFI between 0.90 and 0.95 (Hu and Bentler 1995). To illustrate interactions, simple slope analyses were conducted at low and high levels of racial stress; low levels of racial stress were centered at zero (as one standard deviation below the mean is a negative value and out of the range of the variable) and high levels were one standard deviation above the mean.

## 3 Results

Preliminary analyses, including descriptive statistics (e.g., means and SDs) and bivariate correlations, are in Table S1. Bivariately, maternal racial stress was related to lower social support and higher preparation for bias, cultural socialization, and promotion of mistrust. Child EF was negatively related to maternal preparation for bias and promotion of mistrust, but not maternal social support, racial stress, or cultural socialization.

The path model (Table 1) had a good fit to the data, χ^2^(57) = 105.72, *p* < 0.001; RMSEA = 0.044 (CI_.90_ = 0.031, 0.057); CFI = 0.969. SRMR = 0.037. Mothers used more promotion of mistrust with girls (*β* = 0.09, *p* = 0.049), and girls performed better on the EF tasks than boys (*β* = 0.19, *p* < 0.001). Mothers with more years of education also had children showing better EF scores (*β* = 0.19, *p* = 0.001).

**TABLE 1:** Path model results.

	Preparation for bias				Cultural socialization				Promotion of mistrust				Child executive functioning			
	*B*	SE	*t*	*β*	*B*	SE	*t*	*β*	*B*	SE	*t*	*β*	*B*	SE	*t*	*β*
Social support	**0.05**	**0.02**	**2.36** *	**0.13**	**0.08**	**0.04**	**2.24** *	**0.11**	**0.03**	**0.02**	**2.32** *	**0.12**	0.01	0.02	0.72	0.04
Racial stress (RS)	**0.01**	**0.00**	**2.88** **	**0.23**	**0.01**	**0.00**	**4.97** ***	**0.33**	**0.00**	**0.00**	**2.27** *	**0.23**	0.00	0.00	0.27	0.01
Social support × RS	0.00	0.00	−0.23	−0.01	**0.01**	**0.00**	**2.66** **	**0.17**	0.00	0.00	1.01	0.08	0.00	0.00	0.02	0.00
Preparation for bias	—	—	—	—	—	—	—	—	—	—	—	—	0.00	0.06	−0.05	0.00
Cultural socialization	—	—	—	—	—	—	—	—	—	—	—	—	−0.01	0.03	−0.45	−0.02
Promotion of mistrust	—	—	—	—	—	—	—	—	—	—	—	—	**−0.19**	**0.08**	**−2.47** *	**−0.15**
Prep. bias × RS	—	—	—	—	—	—	—	—	—	—	—	—	**−0.01**	**0.00**	**−3.01** **	**−0.26**
Cult. soc. × RS	—	—	—	—	—	—	—	—	—	—	—	—	0.00	0.00	0.90	0.04
Prom. mistrust × RS	—	—	—	—	—	—	—	—	—	—	—	—	**0.01**	**0.01**	**2.36** *	**0.22**
Recruitment site	−0.04	0.04	−1.01	−0.03	0.18	0.16	1.14	0.07	−0.03	0.02	−1.09	−0.02	0.20	0.05	3.83***	0.15
Child sex	0.04	0.02	1.72	0.07	0.04	0.04	0.96	0.04	0.03	0.02	1.97*	0.09	0.09	0.02	3.91***	0.19
Birth order	0.02	0.02	0.73	0.03	−0.01	0.04	−0.15	−0.01	0.00	0.02	−0.21	−0.01	0.00	0.02	−0.05	0.00
Income‐to‐needs ratio	0.00	0.01	−0.32	−0.01	0.02	0.03	0.81	0.06	−0.01	0.01	−1.30	−0.06	0.01	0.02	0.44	0.04
Married	−0.04	0.03	−1.39	−0.08	−0.01	0.05	−0.29	−0.01	−0.02	0.02	−0.87	−0.05	−0.02	0.03	−0.57	−0.03
Cohabiting	0.02	0.04	0.51	0.03	0.01	0.06	0.11	0.00	0.01	0.02	0.50	0.02	−0.03	0.03	−0.83	−0.04
Education	−0.01	0.01	−1.83	−0.09	0.01	0.01	1.52	0.06	0.00	0.00	−0.36	−0.02	0.02	0.01	3.33***	0.19
Employment	−0.02	0.03	−0.87	−0.05	−0.02	0.05	−0.30	−0.02	0.01	0.02	0.30	0.02	0.01	0.03	0.49	0.03
Maternal age	0.00	0.00	0.83	0.06	0.00	0.00	−0.21	−0.01	0.00	0.00	0.73	0.04	0.00	0.00	−0.25	−0.01

*Note:* Recruitment site: 0 = *NC*, 1 = *PA*. Child sex: 1 = *girl*, 0 = *boy*. Birth order: 1 = *firstborn, 0 = not firstborn*. Married: 1 = *married*, 0 = *not married*. Cohabiting: 1 = *non‐married cohabitating*, 0 = *not cohabitating*. Maternal employment: 1 = *employed*, 0 = *not employed*. Bold values indicate statistically significant results among key study variables.

* *p* < 0.05.

** *p* < 0.01.

*** *p* < 0.001.

**FIGURE 1:**
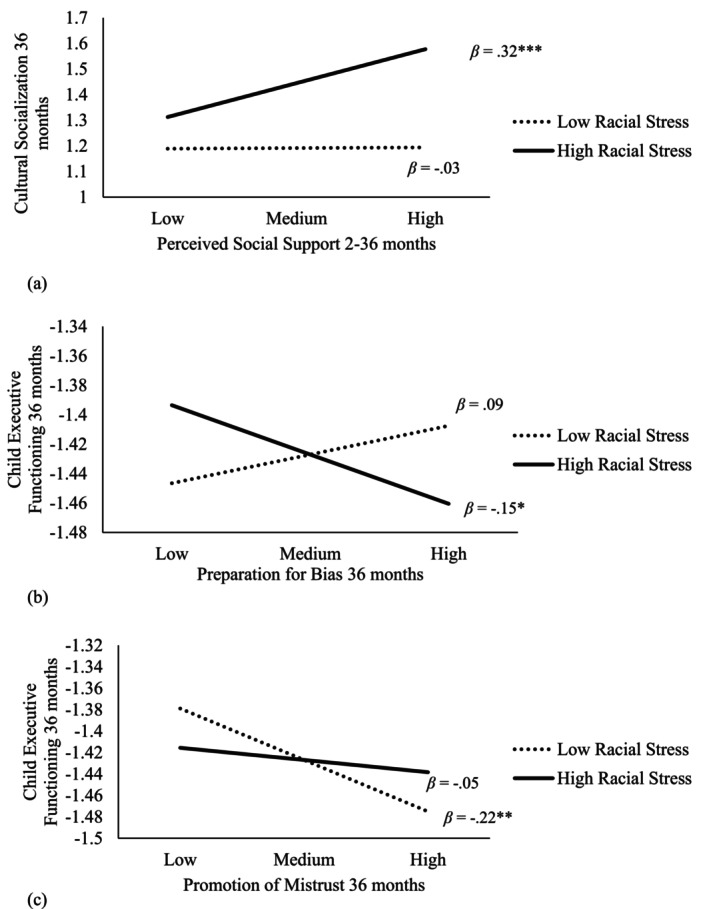
The interaction plots. (a) The interaction between perceived social support and racial stress predicting cultural socialization. (b) The interaction between preparation for bias and racial stress predicting child executive functioning. (c) The interaction between promotion of mistrust and racial stress predicting child executive functioning. *p < 0.05; **p < 0.01; ***p < 0.001.

Perceived social support over 3 years was related to more frequent use of preparation for bias (*β* = 0.13, *p* = 0.02) and promotion of mistrust (*β* = 0.12, *p* = 0.02). Racial stress was also related to more frequent use of preparation for bias (*β* = 0.23, *p* = 0.004) and higher promotion of mistrust (*β* = 0.23, *p* = 0.02). Additionally, an interaction emerged such that racial stress intensified the positive association between perceived social support and cultural socialization. Specifically, under higher levels of racial stress, perceived social support predicted more cultural socialization (*β* = 0.32, *p* = 0.001), whereas this association was not significant under lower levels of racial stress (*β* = −0.03, *p* = 0.62; Figure 1a).

As to child EF, an interaction indicated that racial stress exacerbated the negative link between preparation for bias and child EF; preparation for bias predicted lower child EF under higher racial stress (*β* = −0.15, *p* = 0.02) but not under lower racial stress (*β* = 0.09, *p* = 0.32; Figure 1b). In addition, racial stress reduced the negative link between promotion of mistrust and child EF; parental promotion of mistrust predicted lower child EF under lower racial stress (*β* = −0.22, *p* = 0.004) but not under higher racial stress (*β* = −0.05, *p* = 0.40; Figure 1c). Maternal cultural socialization was not associated with child EF.

## 4 Discussion

The current study examined maternal social support, racial stress, racial socialization strategies, and preschoolers' EF among low‐income, rural Black families. Findings support the RECAST framework (Anderson and Stevenson 2019) and highlight the effects of maternal racial stress on the development of child EF. This study has important implications for the theorization of maternal racial stress and clinical intervention with families experiencing high racial stress.

Supporting our first research hypothesis, both racial stress and social support were positively related to maternal preparation for bias and promotion of mistrust, consistent with previous findings (e.g., McNeil Smith et al. 2016; Saleem et al. 2020). In addition, this study found that maternal perceived social support predicted more cultural socialization only under higher levels of racial stress. The pattern that in the context of high stress, the effect of coping resources becomes more salient aligns with several other studies. Saleem et al. (2016) also found that when racial discrimination was high, neighborhood cohesiveness was linked to a reduced promotion of mistrust among Black parents. Additionally, elevated racial discrimination amplified the positive relations between racial identity and cultural socialization among Black mothers (Holloway and Varner 2021) and strengthened the connection between cultural socialization attitudes and behaviors among Latinx mothers (Derlan et al. 2018). It may appear that social support provides a connection to one's culture and heritage for Black mothers, and in the context of high risk (high racial stress), they may feel extra motivated to turn this into socialization behaviors (Derlan et al. 2018). It is also likely that social support serves as a coping resource that Black mothers may utilize to buffer against racial stress and engage in promoting positive messages about their racial group, which is suggested by the RECAST (Anderson and Stevenson 2019). This is especially important considering the positive effects of cultural socialization on children (e.g., Aguayo et al. 2021; Williams et al. 2023).

Supporting our second hypothesis, maternal racial stress strengthened the link between preparation for bias and lower child EF. Only under high maternal racial stress was preparation for bias related to lower child EF, highlighting maternal racial stress as a contextual stressor for the children, as suggested by the RECAST (Anderson and Stevenson 2019). This finding aligns with Osborne et al.'s (2021) study on Black first‐graders and Espinoza's (Espinoza et al. 2016) study on Mexican American adolescents. In both studies, preparation for bias was not related to child outcomes on average, but turned into an undesired strategy when the environmental risk (parental racial stress) was high. In the current study, observing maternal stress and receiving messages about biases together may confirm each other, which dysregulates the stress response system and disrupts children's neurodevelopment, thus impacting EF (Barbee et al. 2025). As a result, their ability to focus, regulate emotions, and process information may be compromised. This also explains the mixed findings on the effects of this socialization strategy (Espinoza et al. 2016; Evans et al. 2012) and highlights that parents need to tailor their racial socialization strategies according to the contextual risks as well as children's developmental stages.

Contrary to our second hypothesis, maternal racial stress weakened the relation between parental promotion of mistrust and lower child EF. That is, maternal promotion of mistrust was linked to lower child EF in the context of *lower* but not higher racial stress. Although this finding is unexpected, it aligns with the current literature that parental promotion of mistrust was generally related to less optimal socioemotional outcomes in children and adolescents (e.g., Espinoza et al. 2016; Kwon et al. 2022; Saleem et al. 2023) and revealed a possible explanation underlying these findings that this socialization strategy may have comprised children's ability to focus, self‐regulate, and exhibit socially adaptive behaviors as early as in the preschool age. This finding also adds to previous research that the negative effect of this strategy can be reduced if the environmental risk is high, such as when they experience frequent discriminatory experiences based on their race, expanding the RECAST (Anderson and Stevenson 2019) to add contextual and developmental sensitivity. Although this finding may not align with previous findings where researchers found parental promotion of mistrust was related to worse mental health outcomes in the context of higher risks among Mexican American adolescents (Espinoza et al. 2016; Park et al. 2020), it could be age‐specific. Preschool‐aged children may not yet comprehend the nuanced meaning of race, as their cognitive processing tends to be dichotomous. As a result, straightforward messages such as “do not trust individuals from other groups” may be more easily understood than more complex statements like “some individuals from outside groups may be good, but it is important to learn to differentiate them.” Straightforward messages may be particularly easy on preschoolers' cognitive load and EF development when navigating the complex social world. This hypothesis warrants further empirical investigation and underscores the importance of examining the effects of racial socialization strategies in younger populations.

### 4.1 Theoretical Integration

Findings of the current investigation support the RECAST framework (Anderson and Stevenson 2019) by first revealing maternal perceived social support as a key resource factor in effectively managing experienced racial stress and providing more positive socialization messages to their children. Second, this study reveals that the effects of racial socialization practices on children depend on the contextual stress levels. Findings pinpoint important contexts where racial socialization may be more or less effective and thus have significant implications for designing cost‐effective, context‐sensitive intervention efforts.

This study additionally provided nuanced findings regarding the developmental appropriateness of different racial socialization strategies and extended our current understanding of racial socialization to a younger population. In particular, preparation for bias may be beneficial to adolescents as it prepares them to cope and allows them to attribute discriminatory experiences to others' bias (Evans et al. 2012). However, it may be difficult for preschoolers to grasp the complexity of this idea. On the other hand, contrary to findings in adolescents (e.g., Espinoza et al. 2016; Park et al. 2020), the negative effects of parental promotion of mistrust may weaken for preschoolers in the high‐risk context, given the straightforward nature of this strategy. Together, the effectiveness of racial socialization strategies may depend on the context and the developmental stage of children, and a better understanding of these factors can guide future research and intervention efforts.

### 4.2 Strengths, Limitations, and Implications

This longitudinal, multi‐method investigation has several strengths. This study offers great contributions to our current understanding of racial socialization practices in low‐income Black families. Guided by the overarching framework of RECAST (Anderson and Stevenson 2019), this study taps particularly into important questions about racial socialization from a view of resource‐stress interaction. This study additionally extends the current understanding of the developmental appropriateness of different racial socialization strategies to a younger population and to a core regulatory ability, EF.

Findings from the current study need to be interpreted with several limitations in mind. First, as suggested by other researchers (e.g., Umaña‐Taylor and Hill 2020), most racially minoritized families face racial stress, but the particular presentation of stressors may differ based on the race/ethnicity, the context, the geographical location, and the resources that they have. Due to this, current findings may not be generalized to other minoritized families beyond the studied population, and future studies should consider investigating the associations of current study variables among other minoritized families. Second, we examined mothers as they typically serve as the primary socializers in the family (Holloway and Varner 2021). However, evidence suggests Black fathers may have different racial socialization strategies based on the discriminatory experience that they have had based on their race and gender (e.g., Holloway and Varner 2021; McNeil Smith et al. 2016). Findings may thus not be generalizable to fathers or other socializers in the family. Third, we did not control for maternal EF in this study due to a lack of available data. Past research has revealed the association between racial stress and adult EF (e.g., Chen et al. 2022) and parent‐to‐child transmission of EF (e.g., Korucu et al. 2020). It is likely that maternal racial stress can undermine maternal EF, which then links to lower child EF levels, and this hypothesis needs to be tested in future studies. Fourth, we used multi‐timepoint data for social support, which allowed us to reduce measurement error and generate a more stable, trait‐like indicator across early childhood, rather than relying on a single cross‐sectional snapshot (e.g., Conger et al. 2000). However, because other variables were only collected once, and given the secondary analysis nature of the current study, we could not use other assessment points. Future studies should explore the longitudinal, time‐lagged associations among the study variables. Moreover, although the 5‐year racial stress measure reflects salient, chronic exposure relevant to parenting behavior, it began prior to the child's birth and may have involved experiences that could impact children differently than those occurring during their lifetime. Research based on the fetal programming hypothesis suggests that racial stress experienced by mothers during pregnancy can alter fetal brain development, particularly areas involved in EF, and this biologically embedded mechanism can be different from socially/contextually embedded risks, such as postnatal maternal stress (e.g., Barbee et al. 2025). This hypothesis may be explored by future studies. Additionally, the moderate reliability of the promotion of mistrust subscale was likely due to its briefness and the controversy of this socialization strategy. For example, some parents may endorse high scores on low‐mistrust items, such as “Told the child to be careful around other races,” but low scores on high‐mistrust items, like “Told the child other races' negative qualities.” The divergence of responses within the same scale may have affected its reliability. Thus, it warrants future research to examine item‐level patterns or explore different operationalizations of this assessment. The moderate reliability may also have attenuated the observed associations, particularly with child EF under different levels of racial stress, and future studies should replicate this finding. Future studies should also consider differentiating the effects of low‐mistrust versus high‐mistrust messages.

The findings of the current study have several implications. Along with other evidence suggesting context‐sensitive racial socialization strategies (Espinoza et al. 2016; Osborne et al. 2021), the findings of this study may suggest that the effectiveness of racial socialization strategies is dependent on the contextual risk (such as maternal experiences of racial stress), and parents may adjust their strategies to use more straightforward language with preschoolers when the risk is high versus low. This study additionally offers insight into Black mothers' choices of racial socialization strategies and suggests that building strong social support networks may help these mothers to be more in touch with their cultural heritage and promote a sense of cultural pride with their children in buffering the contextual stress. Clinicians working with minoritized families experiencing racial stress should also consider the contextual risk to tailor coping strategies against racism in a context‐sensitive manner. Together, through collaborative efforts to reduce racial discrimination and parental socialization of racially relevant information, children of minoritized racial and ethnic groups can hopefully thrive in a multi‐racial society, and a better understanding of factors promoting the success of racial socialization is just the first step.

## Conflicts of Interest

The authors declare no conflicts of interest.

## Supporting information


**Appendix S1:** famp70068‐sup‐0001‐AppendixS1.docx.

## Data Availability

The data of this study are openly available in the Inter‐university Consortium for Political and Social Research at https://www.icpsr.umich.edu/web/ICPSR/studies/39203/versions/V2.
