# Supplementary material for: Racial Stress, Social Support, and Racial Socialization Among Rural Black Mothers: Associations With Preschoolers' Executive Functioning
Source: Fam Process. 2025 Aug 31;64(3):e70068. doi: 10.1111/famp.70068 (PMC12399874; doi:10.1111/famp.70068)
Supplement: Supplementary file 1 — Appendix S1: famp70068‐sup‐0001‐AppendixS1.docx. [file FAMP-64-0-s001.docx]

**Table S1**

*Descriptive Statistics and Bivariate Correlations*

|  | 1 | 2 | 3 | 4 | 5 | 6 | 7 | 8 | 9 | 10 | 11 | 12 | 13 | 14 | 15 |
| --- | --- | --- | --- | --- | --- | --- | --- | --- | --- | --- | --- | --- | --- | --- | --- |
| 1. Recruitment Site |  |  |  |  |  |  |  |  |  |  |  |  |  |  |  |
| 1. Child Sex | -.07 |  |  |  |  |  |  |  |  |  |  |  |  |  |  |
| 1. Birth Order | .01 | -.02 |  |  |  |  |  |  |  |  |  |  |  |  |  |
| 1. Married | -.05 | -.02 | -.19^**^ |  |  |  |  |  |  |  |  |  |  |  |  |
| 1. Cohabiting | .06 | .01 | .03 | -.27^**^ |  |  |  |  |  |  |  |  |  |  |  |
| 1. Employment | -.06 | -.06 | .05 | .07 | -.06 |  |  |  |  |  |  |  |  |  |  |
| 1. Maternal Age | .06 | .01 | -.33^**^ | .30^**^ | -.13^**^ | .05 |  |  |  |  |  |  |  |  |  |
| 1. Education | -.07 | .02 | .02 | .24^**^ | -.12^*^ | .35^**^ | .24^**^ |  |  |  |  |  |  |  |  |
| 1. Income-to-Needs Ratio | -.05 | -.05 | .06 | .26^**^ | .04 | .46^**^ | .22^**^ | .37^**^ |  |  |  |  |  |  |  |
| 1. Social Support | -.07 | -.11^*^ | .06 | .23^**^ | -.13^**^ | .12^*^ | .10^*^ | .14^**^ | .15^**^ |  |  |  |  |  |  |
| 1. Preparation for Bias | .01 | .08 | .04 | -.10^*^ | .01 | -.08 | .02 | -.08 | -.06 | .04 |  |  |  |  |  |
| 1. Cultural Socialization | .08 | .05 | .00 | .05 | -.03 | .04 | .04 | .12^*^ | .10^*^ | .07 | .37^**^ |  |  |  |  |
| 1. Promotion of Mistrust | -.02 | .09 | .00 | -.07 | -.01 | -.03 | .04 | -.02 | -.05 | .05 | .64^**^ | .25^**^ |  |  |  |
| 1. Racial Stress | .11^*^ | .04 | -.02 | .07 | -.03 | .00 | .08 | .11^*^ | .11^*^ | -.11^*^ | .21^**^ | .31^**^ | .20^**^ |  |  |
| 1. Executive Functioning | .10 | .17^**^ | .01 | .05 | -.04 | .11^*^ | .05 | .20^**^ | .11^*^ | .04 | -.13^*^ | -.01 | -.16^**^ | .02 |  |
| *N* | 437 | 437 | 437 | 437 | 437 | 437 | 437 | 437 | 437 | 437 | 427 | 427 | 427 | 427 | 384 |
| Min | 0 | 0 | 0 | 0 | 0 | 0 | 17 | 7 | 0.00 | 1.50 | 1.00 | 1.00 | 1.00 | 0.00 | -2.16 |
| Max | 1 | 1 | 1 | 1 | 1 | 1 | 47 | 21 | 11.41 | 5.00 | 3.00 | 4.00 | 2.50 | 68.00 | -0.79 |
| Mean | -- | -- | -- | -- | -- | -- | 27.35 | 14.34 | 1.26 | 3.82 | 1.07 | 1.29 | 1.05 | 6.93 | -1.44 |
| *SD* | -- | -- | -- | -- | -- | -- | 5.43 | 2.48 | 1.20 | 0.65 | 0.24 | 0.47 | 0.18 | 11.19 | 0.22 |

*Note*. Recruitment Site: 0 = *NC*, 1 = *PA*. Child Sex: 1 = *girl*, 0 = *boy.* Birth Order: 1 = *firstborn, 0 = not firstborn.* Marital status: 1 = *married*, 0 = *not married*. Cohabitation: 1 = *non-married cohabitating*, 0 = *not cohabitating*. Maternal employment: 1 = *employed*, 0 = *not employed*.

* *p* < .05. ** *p* < .01.
